# Evaluation of Nicotine Dependence Among Smokeless Tobacco Users Using the Fagerstrom Nicotine Dependence Scale for Smokeless Tobacco

**DOI:** 10.7759/cureus.38639

**Published:** 2023-05-06

**Authors:** Hiremath Shaashi uday, Ravikumar Pethagounder Thangavelu, Karthik Rajaram Mohan, Saramma Mathew Fenn, Kumar Appusamy

**Affiliations:** 1 Department of Oral Medicine and Radiology, Vinayaka Mission's Sankarachariyar Dental College, Vinayaka Mission's Research Foundation, Salem, IND; 2 Department of Oral Medicine and Radiology, Vinayaka Mission’s Sankarachariyar Dental College, Vinayaka Mission’s Research Foundation, Salem, IND

**Keywords:** smokeless tobacco (st), hans chewers, panmasala, gutka, fagerstrom nicotine dependence

## Abstract

Nicotine dependence is a current indwelling and challenging health burden among smokeless tobacco users as it involves the compulsive use of a substance despite its known harmful effects. The evaluation of nicotine dependence is challenging as it involves physical and psychological dependence due to nicotine in smokeless tobacco.

Aim and objective: The primary aim is to assess the nicotine dependence in a group of smokeless tobacco by using a six-question Fagerstrom Test for Nicotine Dependence for Smokeless Tobacco (FTND-ST) and to assess the nicotine dependence among three groups, namely Group - 1, who were exclusively pan masala, gutka chewers, Group - 2, who were exclusively Hans users, and Group - 3, who were exclusively betel quid with smokeless tobacco chewers.

Materials and methods: Only those who use smokeless tobacco in the age groups between 21 to 70 years were randomly selected. The total sample size is 100 patients. The age groups were divided into 21-28, 29-35, 36-42, 43-49, 50-56, 57-63, and 64-70. Informed consent was obtained from the participants of the study.

Results: The Hans chewers are predominantly females. Pan masala and gutka chewers are predominantly males.

Conclusion: Smokeless tobacco chewers like pan masala were found to have high mean nicotine dependence Fagerstrom score than Hans and betel quid with smokeless tobacco chewers.

## Introduction

One of the common problems we are currently facing in the world is the consumption of smokeless tobacco. Smoking is not only injurious to oneself but also to the surrounding people. Moreover, it has adverse ill effects on various body parts, which can lead to worse situations when out of control. Although smoking is the most common problem in India, we will focus on smokeless tobacco in this study. While measures of nicotine dependence have been developed and validated for cigarette smokers, few nicotine dependence measures have been assessed and evaluated for research and clinical use among smokeless tobacco users [1]. Smokeless tobacco is a tobacco product used by means other than smoking. Their use involves chewing, snuffing, or placing the product between gums, cheeks, or lips. Even though smokeless tobacco has fewer chemicals, it does not mean the user will quit using it. The tobacco and nicotine content in it causes tobacco dependence, and the user finds it hard to resist nicotine. Smokeless tobacco will mainly cause oral problems to the user, such as leukoplakia, and oral cancer; long-term use increases the risk of mortality due to heart diseases, such as the increased risk for coronary atherosclerosis and stroke. Oral and mouth cancer can affect the pharynx (throat) and larynx. Smokeless tobacco can cause various dental problems like gingival recession, periodontitis, abrasion, gingivitis, stains, etc. It will also lead to bad breath (halitosis). Cardiovascular problems can also occur due to the nicotine in smokeless tobacco that goes into the bloodstream. While a majority of research involving smokeless tobacco has focused on the prevalence and epidemiological investigations, a few reports have assessed the dependence-producing potential of tobacco products [2,3].

## Materials and methods

The study was approved by Institutional Reviewer BoardVinayaka Mission's Sankarachariyar Dental College, Vinayaka Mission's Research Foundation, Salem, India with approval IRC/041617022022/S/4. The study participants were the randomly selected dental outpatients who visited the outpatient department between January to September 2022 and had the habit of chewing tobacco for at least a minimum period of three years. Nicotine dependence among smokeless tobacco users was assessed by using the Fagerström Test for Nicotine Dependence for Smokeless Tobacco (FTND-ST) scale by face to face questionnaire among three Groups, namely Group 1 - those who chew pan masala or gutka, Group 2 - those who chew Hans, and Group 3 - those who chew betel quid with smokeless tobacco. The inclusion and exclusion criteria for this study among smokeless tobacco users are listed in Table 1.

**Table 1:** Inclusion and exclusion criteria

Inclusion criteria	Exclusion criteria
a) Participants who are current users of smokeless tobacco for not less than three years	a) Patients who use smoking forms of tobacco such as cigarette, bidi, cheroot, pipe, etc.
b) Smokeless tobacco users between 21 to 70 years of age	b) Patients who are not smokers
	c) Patients less than 21 years and older adults above 70 years
	d) Teenagers of the age group between 15 to 17 years
	e) Patients with smokeless tobacco chewing for one- or two-year duration
	f) Smokeless forms of tobacco typically taken through nostrils, such as dry snuff, creamy, moist snuff, and usually dipped and sweetened tobacco, such as snus, were not included in this study
	g) Bedridden or terminally ill cancer patients
	h) Patients who were not willing to participate or provide written informed consent for the study

## Results

**Figure 1:**
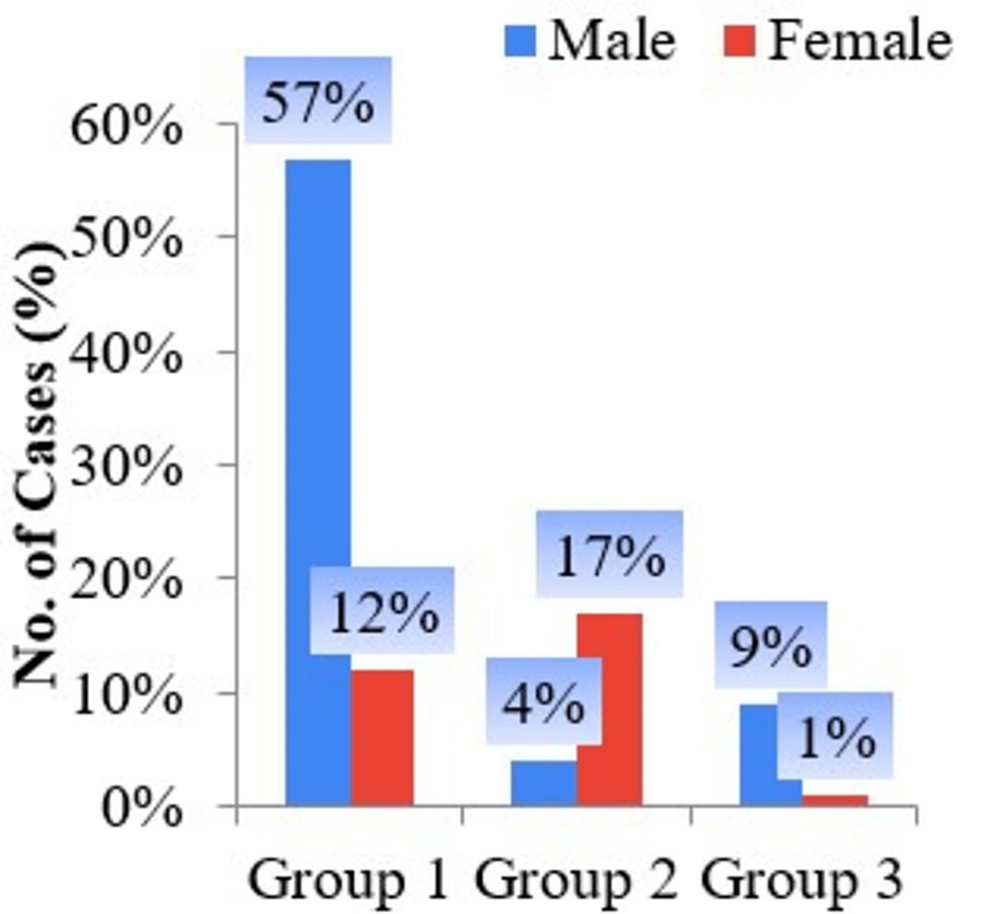
Bar diagram of groups and gender

Among the 100 smokeless tobacco chewers, 70 were males and 30 were females. In Group I, 57% were males and 12% were females. In Group II, females were more predominant (17%) as compared to males (4%). Group III comprised about 9% males and 1% females (Figure 1).

**Figure 2:**
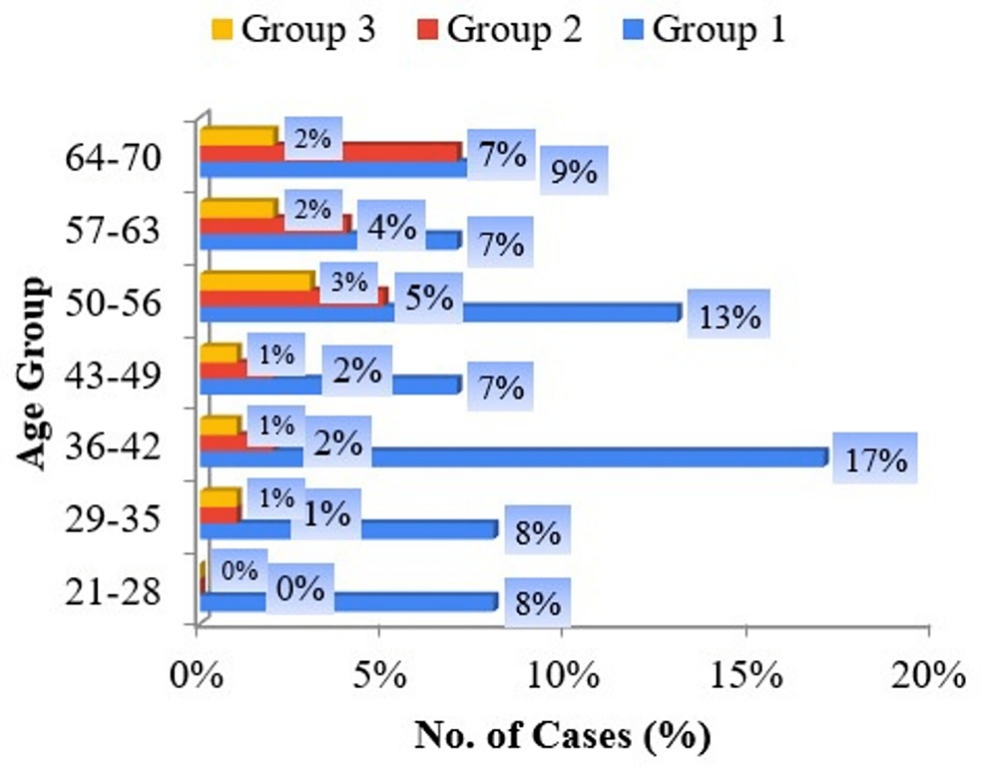
Bar diagram of age groups

The predominant subgroup which uses pan masala and gutka comprised males in the age group 36-42 years. The predominant subgroup which uses Hans comprised females in the age group 64-70 years. The predominant age group which uses betel quid with smokeless tobacco comprised 50-56-year-olds (Figure 2).

The distribution of smokeless tobacco chewers in FTND-ST is shown in Table 2.

**Table 2:** Distribution of smokeless tobacco chewers in the Fagerstrom Test for Nicotine Dependence for Smokeless Tobacco

Fagerstrom Test for Nicotine Dependence for Smokeless Tobacco (FTND-ST)		N	%
How soon after you wake up do you place your first dip?	After 60 min	13	13.0%
	31 - 60 min	21	21.0%
	6 - 30 min	58	58.0%
	Within 5 min	8	8.0%
	Total	100	100.0%
How often do you intentionally swallow tobacco juice	Never	64	64.0%
	Sometimes	34	34.0%
	Always	2	2.0%
	Total	100	100.0%
Which chew would you hate to give up most?	All others	42	42.0%
	The first one in the morning	58	58.0%
	Total	100	100.0%
How many cans/pouches per week do you use?	1	7	7.0%
	2 -3	33	33.0%
	More than 3	60	60.0%
	Total	100	100.0%
Do you chew more frequently during the first hours after awakening than during the rest of the day?	No	38	38.0%
	Yes	62	62.0%
	Total	100	100.0%
Do you chew if you are so ill that you are in bed most of the day?	No	75	75.0%
	Yes	25	25.0%
	Total	100	100.0%

The distribution of FTND-ST among various groups, namely Group I, Group II, and Group III smokeless tobacco users, is depicted in Table 3.

**Table 3:** The distribution of the Fagerstrom Test for Nicotine Dependence for Smokeless Tobacco (FTND-ST) among groups of smokeless tobacco chewers

FTND-ST		Group 1		Group 2		Group 3	
		Count	Table N %	Count	Table N %	Count	Table N %
How soon after you wake up do you place your first dip?	After 60 min	9	9.0%	4	4.0%	0	0.0%
	31 - 60 min	13	13.0%	5	5.0%	3	3.0%
	6 - 30 min	44	44.0%	10	10.0%	4	4.0%
	Within 5 min	3	3.0%	2	2.0%	3	3.0%
How often do you intentionally swallow tobacco juice?	Never	41	41.0%	18	18.0%	5	5.0%
	Sometimes	26	26.0%	3	3.0%	5	5.0%
	Always	2	2.0%	0	0.0%	0	0.0%
Which chew would you hate to give up most?	All others	28	28.0%	10	10.0%	4	4.0%
	The first one in the morning	41	41.0%	11	11.0%	6	6.0%
How many cans/pouches per week do you use?	1	5	5.0%	2	2.0%	0	0.0%
	2 -3	22	22.0%	6	6.0%	5	5.0%
	More than 3	42	42.0%	13	13.0%	5	5.0%
Do you chew more frequently during the first hours after awakening than during the rest of the day?	No	28	28.0%	8	8.0%	2	2.0%
	Yes	41	41.0%	13	13.0%	8	8.0%
Do you chew if you are so ill that you are in bed most of the day?	No	50	50.0%	20	20.0%	5	5.0%
	Yes	19	19.0%	1	1.0%	5	5.0%

**Figure 3:**
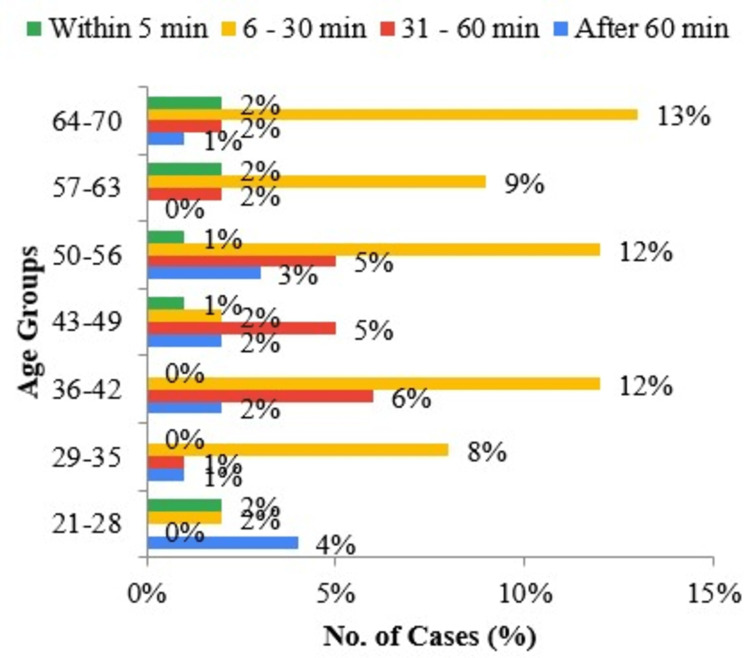
Bar diagram of age groups according to the answers to “How soon after you wake up do you place your first dip?”

Most of the smokeless chewers chew their tobacco as soon as they wake up and place their first dip for a minimum period of 5-30 minutes (Figure 3).

**Figure 4:**
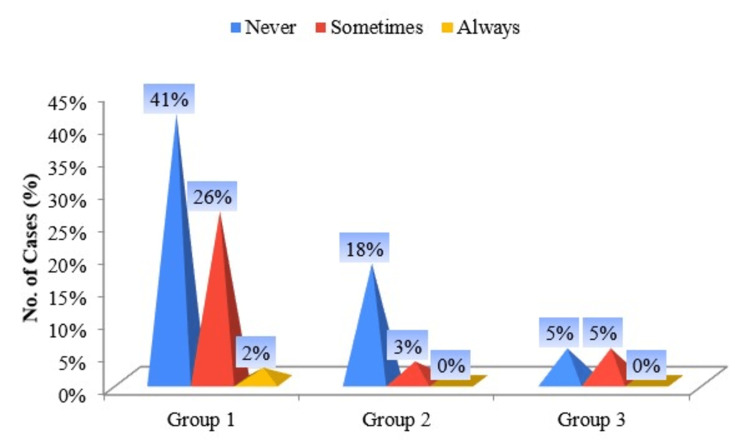
Pyramid diagram of groups according to the answers to “How often do you intentionally swallow tobacco juice?”

Most of the male smokeless tobacco users of pan masala and gutka do not intentionally swallow tobacco juices, whereas Hans chewers among females sometimes swallow tobacco juices (Figure 4).

**Figure 5:**
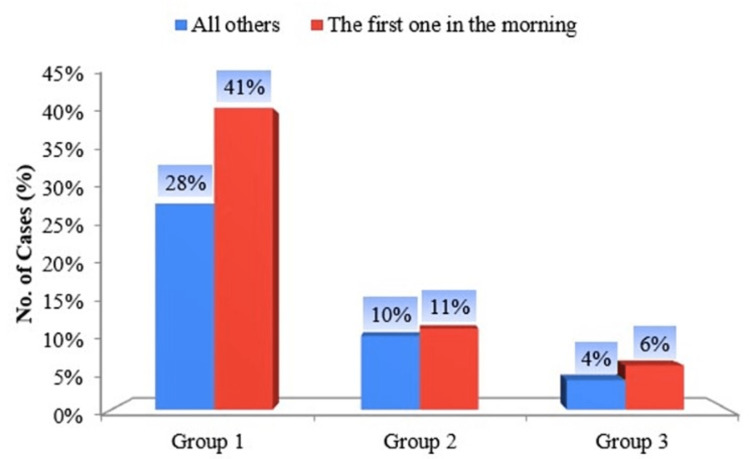
Bar diagram of groups according to the answers to “Which chew would you hate to give up most?”

Compared to male chewers, females chew their smokeless tobacco as soon as they wake up in the morning. As much as 41% were not willing to quit their pan masala and gutka tobacco chewing habit in the morning in Group I, 11% of females were not ready to leave the practice of Hans chewing in Group II, and 6% of females were not willing to quit their habit of betel quid along with smokeless tobacco products in Group III (Figure 5).

**Figure 6:**
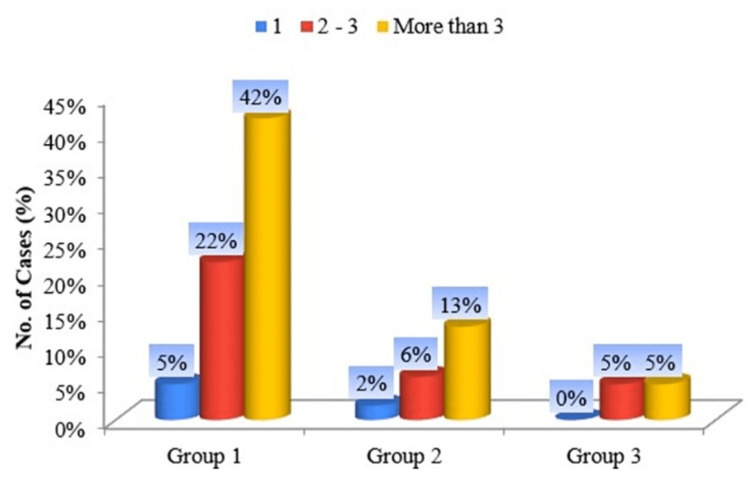
Cylinder diagram of groups according to “How many cans/pouches per week do you use?”

The majority of smokeless tobacco chewers use smokeless tobacco products at least three times a day (Figure 6).

**Figure 7:**
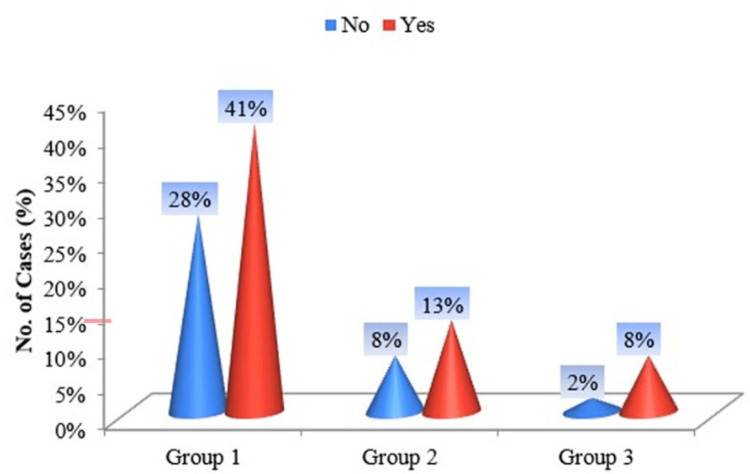
Cone diagram of groups according to "Do you chew more frequently during the first hours after awakening than during the rest of the day?”

The majority of female smokeless tobacco users chew their tobacco within the first hour of waking up than during the rest of the day (Figure 7).

**Figure 8:**
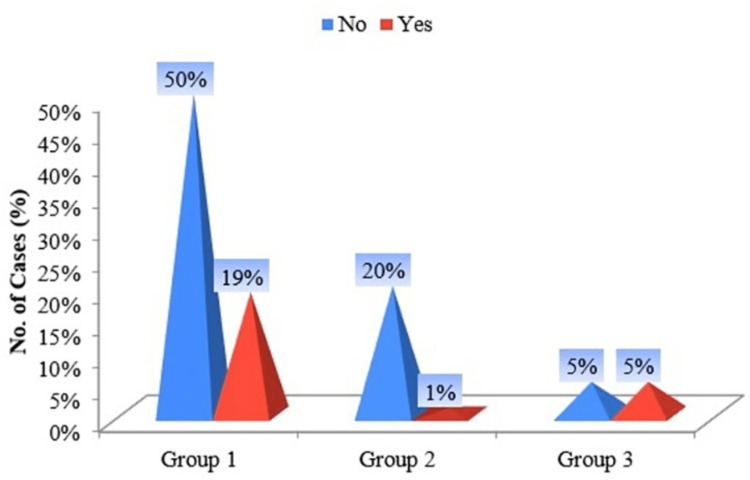
Pyramid diagram of groups according to "Do you chew if you are so ill that you are in bed most of the day?”

Most female smokeless tobacco users of pan masala and gutka among Group I chew their tobacco in bed despite their illness most of the day (Figure 8).

The comparison of the FTND-ST among groups by chi-square test is shown in Table 4.

**Table 4:** Comparison of Fagerstrom Test for Nicotine Dependence for Smokeless Tobacco (FTND-ST) among groups * Statistically not significant; ** Statistically significant

FTND-ST	N	Mean	SD	Chi-Square	df	p
How soon after you wake up do you place your first dip?	100	1.61	0.815	61.520	3	0.000**
How often do you intentionally swallow tobacco juice?	100	0.38	0.528	57.680	2	0.000**
Which chew would you hate to give up most?	100	0.58	0.496	2.560	1	0.11
How many cans/pouches per week do you use?	100	1.53	0.627	42.140	2	0.000**
Do you chew more frequently during the first hours after awakening than during the rest of the day?	100	0.62	0.488	5.760	1	0.016*
Do you chew if you are so ill that you are in bed most of the day?	100	0.25	0.435	25.000	1	0.000**

Result

There is a statistically significant difference between “How soon after you wake up do you place your first dip?” (1.61±0.815, p <0.001), “How often do you intentionally swallow tobacco juice?” (0.38±0.528, p <0.001), “How many cans/pouches per week do you use?” (1.53±0.627, p <0.001), “Do you chew more frequently during the first hours after awakening than during the rest of the day?” (0.62±0.488, p <0.05) and “Do you chew if you are so ill that you are in bed most of the day?” (0.25±0.435, p <0.001) in this study.

## Discussion

Smokeless tobacco

A tobacco product used in ways other than smoking (burnt tobacco) is smokeless tobacco that can be chewed, sniffed, or sandwiched between the cheek, lips, or gums [3].

Prevalence of smokeless tobacco in India

The prevalence of smokeless tobacco in India is depicted in Table 4 [3-5].

**Table 5:** Prevalence of use of smokeless tobacco in India

Author	Year	Place	Gender ( M/F) of tobacco chewers
Mehta et al. [3]	1969	Bhavnagar, Gujarat	M = 9, F= 15
Mehta et al. [3]	1969	Darbhanga, Bihar	M = 28, F = 7
Mehta et al. [3]	1969	Ernakulam, Kerala	M = 14, F = 38
Mehta et al. [3]	1969	Srikakulam, Andhra Pradesh	M = 4, F = 3
Mehta et al. [3]	1969	Singhbum, Jharkhand	M = 17 , F = 26
Mehta et al. [3]	1972	Pune, Maharashtra	M = 53, F = 49
Wahi et al. [4]	1968	Mainpuri, Uttarpradesh	M = 21, F = 9
Sankaranarayanan et al. [5]	2000	Trivandrum (urban), Kerala	M = 27, F = 26

Determinants of the use of smokeless tobacco

The use of tobacco is a complex process that is influenced by a wide range of variables, including social, environmental, psychological, and genetic aspects that are associated with tobacco use. Gender, parental use, peer use, exposure to commercials, lack of understanding of the health risks, and peer use are the determinants related to smokeless tobacco use [5]. 

Forms of smokeless tobacco used in South India

The most common forms of smokeless tobacco used in South India are pan masala, gutka, Hans, and betel quid with smokeless tobacco products [5].

Paan (Betel Quid)

Paan, used in many South Asian languages, literally means "leaf." In the betel leaf, several components are wrapped. Tobacco, seeds, quenched slaked lime, spices, areca nut wrapped in betel quid, and quenched lime are all frequent ingredients in paan [5].

Pan Masala

Pan masala is a processed form of tobacco mixed with betel leaves, sweetened rose petal paste, sugar, cardamom, clove, coriander seeds, dried dates, herbs, and amla [5].

Gutkha

The main ingredients in gutka, a type of smokeless tobacco, are powdered tobacco, areca nuts (the fruit of Areca catechu), and slaked lime (aqueous calcium hydroxide). Slaked lime, areca nuts, chewing tobacco, spices, and catechu packaged in tins or pouches make up gutkha [5]. Fine areca nut grains found in gutka, in addition to irritating oral tissues mechanically, also allow ground tobacco to attach to the damaged mucosa, producing morphologic alterations and membrane damage. As a result, areca nuts combined with tobacco may hasten the development of oral submucous fibrosis in people who regularly chew gutka [6].

Chemical ingredients of smokeless tobacco

The following are the chemical ingredients of smokeless tobacco: (i) Tobacco-specific nitrosamines (TSNA), which are produced from tobacco alkaloids during curing, fermentation, and ageing; (ii) N-nitrosamine acids, which are produced from tobacco leaf amino acids that can be N-nitrosated; (iii) volatile N-nitrosamines; (iv) polycyclic aromatic hydrocarbons; (v) volatile aldehydes (formaldehyde, acetaldehyde, carbon aldehyde and (vi) heavy metals: cadmium, uranium and polonium.

Smokeless tobacco products have many of the most potent carcinogens, known as TSNA. Both 4-(methylnitrosamino)-1-(3-pyridyl)-1-butanone (NNK) and N-nitrosonornicotine (NNN) exhibit potent carcinogenic effects.

Chewing betel quid with tobacco was the predominant form of smokeless tobacco use among females older than 35 years in a cohort study in Mumbai, India, and had a high mortality rate [6].

Over a 10-year period, house-to-house surveys in the Ernakulam region of Kerala, India, were used to document the mortality experience of a cohort of 10,287 people aged 15 and over who had been randomly chosen from the population. Age and sex distribution were taken into account as well as the chewing and smoking of tobacco in the analysis of mortality rates. Females were the ones who chewed tobacco most frequently, and these women had considerably higher age-adjusted mortality rates than those who did not (relative risk 1.3) [7-12].

Our study showed that the predominant smokeless tobacco use among females was of Hans and in males, it was of paan or gutkha. Furthermore, there exists a statistically significant correlation among the first dip of users of smokeless tobacco as soon as they awake in the morning, and shows a pattern of an increased number of times of use of smokeless tobacco, swallowing of tobacco juices, and also intake in the bed even during illness.

Oral health consequences of smokeless tobacco

Oral squamous cell carcinoma (SCC) and oral potentially malignant disorders (OPMDs) such as leukoplakia, tobacco pouch keratosis, and oral submucous fibrosis (OSMF) are the most common lesions associated with smokeless tobacco users [13].

Oral Submucous Fibrosis

Constant chewing of Gutka, which contains powdered areca nut, causes microtrauma. The nitrosation of arecoline and arecaidine, alkaloids released from areca nut, causes DNA alkylation and results in the proliferation of fibroblasts. Copper leached into the saliva by areca nut chewing further stimulates fibrogenesis through the upregulation of lysyl oxidase (LOX) activity, resulting in a pale blanched appearance of the affected oral mucosa. Flavonoids and tannins help cross-link collagen fibres in the oral mucosa, resulting in increased fibrogenesis that gradually results in diminished mouth opening (trismus), resulting in a potentially pre-malignant disorder called oral submucous fibrosis.

Ahmad et al. (2006), Saraswathi et al. (2006), Hazarey et al. (2007), and More et al. (2020) stated the increased incidence of oral submucous fibrosis among gutka chewers [14-17].

Oral Cancers

Sapkota et al. (2007) stated an increased risk of hypopharyngeal cancers among gutka chewers [18].

Tobacco Pouch Keratosis and Other Periodontal Problems

A whitish, wrinkled appearance on the vestibule usually occurs in the mandibular, maxillary labial, and buccal vestibular regions, where tobacco is frequently pouched, called tobacco pouch keratosis. Bhandarkar et al. stated that tobacco pouch keratosis lesions and periodontal problems such as gingival recession due to loss of attachment, periodontal pocket, and extrinsic stains occur more among Hans than gutka chewers. The increased number of periodontal problems occurs due to the absorption of nicotine alkaloid from smokeless tobacco, which impairs the immune response of T-lymphocytes, phagocytic activity of neutrophils and increases the level of tissue destructing cytokines such as Tumour Necrosis Factor-alpha [19].

Limitations

The participants in this study were unicentric and confined to a particular location, Salem, Tamil Nadu, India. It is not multicentric and does not involve other geographical locations. The study did not assess nicotine dependence among smokeless tobacco users for one or two years. The study did not involve teenagers (15 to 17 years) who can also have high addiction to smokeless forms of tobacco, and their nicotine dependence was not assessed. The study did not assess nicotine dependence among dry snuff-containing powdered tobacco users, typically sniffed through nostrils, and moist, creamy snuff and snus forms of smokeless tobacco, usually dipped. Bias occurs in determining the nicotine dependence among various smokeless tobacco users, such as pan masala, Hans, gutka, and betel quid with smokeless tobacco, as the nicotine content varies among various commercially available smokeless tobacco products used according to their manufacturer brands and geographical locations. Amith et al. (2018) stated that pan masala tobacco chewers had the highest nicotine content (21.70%), whereas cigarettes had the lowest level (17.67%). Cigarettes and pan masala tobacco chewers had average nicotine levels of 7.84 +/- 5.10 and 16.30+/- 3.33, respectively [20]. McAdam et al. (2019) stated that smokeless tobacco products are composite materials with various compositions, such as nicotine, sugars, sodium and chloride ions, water, and humectants. Tobacco is frequently a variable component (30-90%) among smokeless tobacco products [21].

In order to refrain from using smokeless tobacco, distraction abstinence is beneficial to conscious abstinence. As a result, intellectually stimulating activities must be incorporated into de-addiction programmes for smokeless tobacco cessation strategies [22]

## Conclusions

Smokeless tobacco users are increasing among the south Asian population, and the need of the hour is to start more tobacco cessation centres and continue education programs on tobacco cessation and create awareness among the public. Therefore, professionals must ensure that proper education and awareness programs on tobacco cessation and management of nicotine addiction by nicotine replacement therapy are essential and challenging to discourage the use of smokeless tobacco chewing.
